# The New Taxes

**Published:** 1916-04-15

**Authors:** 


### THE NEW TAXES.

These are days in which an authority not less than that of Cæsar Augustus has decreed that all the world shall be taxed. And no citizen need trouble to repair to his own city for the purpose. Where'er he be he may rest, with the certainty that he will be taxed while he waits. The Chancellor deals with millions with an airy grace and the taxpayer suffers in silence, and even in contentment. This latter and unusual phenomenon is generally interpreted as an index of the determination of the country to carry through the war to a successful end, and it thus has its significant aspect. Members of the medical profession take their stand on this point with their fellow-citizens and will pay what is decreed to be their share. They cannot profess to be financial experts and they are certainly not anxious to discover excuses. In short, they, like the rest of the community, will support the new demands with philosophy, and will find what silver bullets are required at their hands. Some of them will doubtless feel that the distribution of burdens might have been more equitable. But all will pay and will endeavour to look pleasant.

The increased income-tax is, of course, a serious matter for members of the profession, as it is for the middle classes generally. The specially severe demands which fall upon incomes above, say, £2,000, can affect but few medical men, and hence we can contemplate them with resignation. The demands below that sum have a personal quality which is for many serious enough in all conscience. In reference to the increased licence duty on motor-cars there will certainly be a word of remonstrance. Many a doctor nowadays cannot possibly do his work without a car, and this consideration has special force in reference to those who are undertaking the practice of an absent colleague. Further, motoring has become much more expensive of recent months in consequence of the high price of petrol, and this is the more irritating in view of the suspicion that the increase is due rather to the greed of the trusts than to ordinary commercial activities. Again, a number of cars popular with the profession just come within the treble-duty limit, as the horse-power is measured by the official standard. Thus a car which is fixed at 16.9-h.p., and on which formerly the duty was six guineas, will now be rated at eighteen guineas, though presumably a medical practitioner will continue to enjoy a reduction of 50 per cent. Even this is a severe demand on what is an essential condition of much professional work. Take along with these penalties the decision to reduce notification fees from half a crown to one shilling, and it is easy to understand that the medical profession learns with composure that the cinematograph has become a more expensive entertainment and that stalls at the opera have risen in price. Sugar and soda-water, coffee and cocoa, with their added charges, are part of the common lot, but it is the income-tax and the new motor duties which will make the most severe inroads on the not overfull pockets of the doctors.

